# Activity Monitor Intervention to Promote Physical Activity of Physicians-In-Training: Randomized Controlled Trial

**DOI:** 10.1371/journal.pone.0100251

**Published:** 2014-06-20

**Authors:** Anne N. Thorndike, Sarah Mills, Lillian Sonnenberg, Deepak Palakshappa, Tian Gao, Cindy T. Pau, Susan Regan

**Affiliations:** 1 Department of Medicine, Massachusetts General Hospital, Boston, Massachusetts, United States of America; 2 Food and Nutrition Services, Massachusetts General Hospital, Boston, Massachusetts, United States of America; Tokyo Institute of Technology, Japan

## Abstract

**Background:**

Physicians are expected to serve as role models for healthy lifestyles, but long work hours reduce time for healthy behaviors. A hospital-based physical activity intervention could improve physician health and increase counseling about exercise.

**Methods:**

We conducted a two-phase intervention among 104 medical residents at a large hospital in Boston, Massachusetts. Phase 1 was a 6-week randomized controlled trial comparing daily steps of residents assigned to an activity monitor displaying feedback about steps and energy consumed (intervention) or to a blinded monitor (control). Phase 2 immediately followed and was a 6-week non-randomized team steps competition in which all participants wore monitors with feedback. Phase 1 outcomes were: 1) median steps/day and 2) proportion of days activity monitor worn. The Phase 2 outcome was mean steps/day on days monitor worn (≥500 steps/day). Physiologic measurements were collected at baseline and study end. Median steps/day were compared using Wilcoxon rank-sum tests. Mean steps were compared using repeated measures regression analyses.

**Results:**

In Phase 1, intervention and control groups had similar activity (6369 vs. 6063 steps/day, p = 0.16) and compliance with wearing the monitor (77% vs. 77% of days, p = 0.73). In Phase 2 (team competition), residents recorded more steps/day than during Phase 1 (Control: 7,971 vs. 7,567, p = 0.002; Intervention: 7,832 vs. 7,739, p = 0.13). Mean compliance with wearing the activity monitor decreased for both groups during Phase 2 compared to Phase 1 (60% vs. 77%, p<0.001). Mean systolic blood pressure decreased (p = 0.004) and HDL cholesterol increased (p<0.001) among all participants at end of study compared to baseline.

**Conclusions:**

Although the activity monitor intervention did not have a major impact on activity or health, the high participation rates of busy residents and modest changes in steps, blood pressure, and HDL suggest that more intensive hospital-based wellness programs have potential for promoting healthier lifestyles among physicians.

**Trial Registration:**

Clinicaltrials.gov NCT01287208.

## Introduction

A growing body of evidence suggests that poor physician health is associated with suboptimal patient care. [1]–[4] “Physician wellness” has typically been described in the context of mental health, [5]–[7] with less attention to the positive aspects of health promotion. [8], [9] Physicians are expected to serve as role models for healthy lifestyles, [10] and evidence suggests that physicians are more likely to counsel when they practice these behaviors themselves. [11]–[14] However, many physicians do not take the time to care for themselves, [6], [15]–[19] and long work hours reduce time for healthy behaviors. [6], [17], [20], [21] Lack of sleep, stress, and intense interactions with patients contribute to burnout, substance abuse, and depression [2], [6], .

Regular physical activity helps prevent obesity and chronic disease [23] and is associated with reduced psychological symptoms, including depression, anxiety, and burnout symptoms. [24]–[27] Residency is a period when time-constraints are particularly acute, and symptoms of burnout and depression are high. [2], [28], [29] One survey of residents found that self-care activities, including exercise, were associated with the absence of burnout. [30] However, residents are less likely to exercise regularly than medical students or attendings, [21], [31] and a survey of radiology residents found that only 37% engaged in recommended guidelines for physical activity. [20] A study of military physicians found that physical fitness declined and weight increased during residency training, [32] and a study of 375 residents from two academic medical centers found that third year medical residents were more likely to be overweight than first year residents [33].

Promoting physical activity among residents could help establish a pattern of healthy lifestyle during and after residency [34] as well as reduce depression and burnout. Physical activity interventions that use pedometers or activity monitors can be effective among healthy, working populations. [35]–[37] We conducted a 2-phase study over 12 weeks to determine if use of an activity monitor as part of a resident wellness program would result in increased physical activity levels. We hypothesized that 1) residents who were randomly assigned to an activity monitor with visual feedback would have higher physical activity levels as measured in steps per day compared to residents assigned to a blinded monitor and 2) conducting a team-based steps competition would increase activity levels of residents compared to the non-competition phase. Phase 1 was a randomized controlled trial, and Phase 2 was a team-based steps competition.

## Methods

### Ethics Statement

This study was approved by the Partners Healthcare Institutional Review Board on June 28, 2010. Written informed consent was obtained from all study subjects upon enrollment. The authors confirm that all ongoing and related trials for this intervention are registered. This study is registered at Clinicaltrials.gov NCT01287208. The study was registered after all subjects were enrolled but prior to the intervention. The registration delay was due to a misunderstanding by the investigator. The study protocol that was registered was exactly the same as the study protocol approved by the Institutional Review Board prior to subject enrollment. Subjects were recruited starting in July 2010 and follow-up was completed in May 2011. The protocol for this trial and supporting CONSORT checklist are available as supporting information; see Checklist S1 and Protocol S1.

### Setting and Participants

Massachusetts General Hospital (MGH) is a 907 bed teaching hospital in Boston, Massachusetts. Since 2005, the hospital has offered a ten week team-based, worksite wellness program called “Be Fit” at no cost to employees, [38], [39] but medical residents have not been able to participate because of their intense work schedules. The current study was developed as a wellness program for residents that would be both convenient and fun and therefore not perceived as burdensome.

During the academic year from July 2010 through June 2011, there were 169 Internal Medicine and Medicine/Pediatric residents employed at the hospital. From July through November 2010, 127 medicine residents participated in a required ambulatory rotation during which the study team conducted a one-hour session as part of the ambulatory curriculum to describe the exercise and nutrition program.

### Exercise and Nutrition Program

During the 12 week study period from January through March 2011, all residents were given access to the on-site fitness center and could complete a one hour personal training session weekly at no cost. To address nutrition, a “Be Fit” lunch prepared by the MGH Food and Nutrition Services was substituted once a week for one of the daily catered resident lunches at their noontime conference. Residents could schedule up to two individual appointments with one of the Be Fit staff nutritionists during the study period.

### Activity Monitor

All study participants were provided with an activity monitor (Fitbit) that tracked daily activity and wirelessly uploaded the data to a website (http://www.fitbit.com). The Fitbit monitors are valid and reliable devices for monitoring step counts in healthy young adults. [40] The device was lightweight (11 grams) and could be worn clipped to the clothing or in a pocket. The monitor displayed steps, energy consumed, and distance traveled, and it also displayed an activity “avatar” that would grow larger with increasing activity and smaller with more sedentary behavior. The website allowed users to track their data over time, log daily weights, and enter dietary information to calculate daily caloric intake.

### Phase 1 Intervention: Randomized Trial

Phase 1 of the intervention started at the beginning of January 2011 and lasted for six weeks. Half of residents were assigned to the activity monitor with visible data (intervention) and half were assigned to an identical activity monitor that was blinded and did not display data (control). Prior to Phase 1, all participants were simultaneously randomized to the intervention or control arm, stratifying by year of training. A random number was assigned to each subject using the built-in random number function in Microsoft Access. A list was created in which subjects were grouped by year of training and then sorted by the random number within each year. Study arm assignments were made by alternating allocation of the listed subjects to intervention or control. The study statistician generated the random allocation sequence. All participants were given a unique anonymous email address that was not connected to their work email and could be used to log on to the Fitbit website to set up an account and provide information on gender, age, height, and weight. Residents randomized to the intervention could log on to the website throughout Phase 1, but residents randomized to control were not able to log on to the website after the account was set up.

Study staff sent weekly emails to remind residents to charge the device and to offer an incentive for wearing it. Residents who wore the monitor at least five of seven days of the week were included in a lottery for a $10 gift card, and two winners were announced in the following week’s email. Study staff monitored the Fitbit website to determine if the blinded monitors were functioning properly. Individual technical assistance was provided to residents as needed.

### Phase 2 Intervention: Non-randomized Team Competition

At the end of Phase 1, residents in the control group were given instructions to unblind their devices so that the steps, calories, and distance would be visible and they could utilize the Fitbit website. Residents were divided into three teams based on year of training: post-graduate year one (PGY-1), post-graduate year two (PGY-2), and post-graduate year three or four (PGY-3). All participants were notified that their team would be competing for the highest average number of steps per week for six weeks. Each week, two residents from the winning team received a $10 gift card.

### Measures and Outcomes

All residents participated in an assessment before and after the 12 week study. Baseline assessments were conducted between July and November 2010, and final assessments were conducted in April and May 2011. We offered four baseline and three follow-up assessment dates so that residents could choose a morning that they would not be post-overnight call. Assessments were performed between 6∶30 am and 8∶00 am so that subjects could fast overnight. Height was recorded at baseline, and weight, waist circumference, blood pressure, and fasting lipids were measured at baseline and the final assessment. Residents completed a survey with questions about demographics, living with a significant other, smoking status, and weight gain or loss over the previous year.

Compliance with wearing the activity monitor was defined as the percentage of days the activity monitor was worn (500 or more steps recorded in a day) during each phase. We defined inpatient and outpatient weeks for each resident by reviewing the electronic resident scheduling system to determine whether the resident was on an inpatient or outpatient (ambulatory) rotation for each week of the study. If the resident switched rotations in the middle of a week, they were assigned to the rotation on which they spent the most number of days for that week.

### Analysis

The baseline differences between the intervention and control groups were assessed with chi-squared and t-tests. An intention-to-treat analysis of steps for the randomized phase was performed using median steps per day because steps were not normally distributed. Days with no steps recorded were counted as 0 steps per day. Compliance was assessed on a daily basis; participants were considered compliant on days when the monitor recorded at least 500 steps. We report the percentage of days that participants were compliant. We tested differences in compliance using logistic regression models adjusted for clustering within participant.

The median steps per day were compared using Wilcoxon rank-sum tests. Analyses of the team competition phase were performed to compare mean steps per day on days when the activity monitor was worn, defined as 500 or more steps recorded in a day. For the analysis of inpatient and outpatient weeks, we excluded vacation days. We used repeated measures regression analyses to assess the effects of study group and rotation (inpatient vs. outpatient) on steps per day when the monitor was worn. We compared physiologic measurements and self-reported minutes of activity at baseline and end of study for all residents who had a measurement from both assessments using t-tests and Wilcoxon rank-sum tests. All analyses were conducted using Stata statistical software (StataCorp, 2008. Stata Statistical Software: Release 10. College Station, TX: Stata Corporation). We calculated a sample size of 50 per group would have 84% power to detect a difference of 1200 steps assuming that the common standard deviation is 2000 steps using a two group t-test with a 0.05 two-sided significance level.

## Results

**Figure 1:**
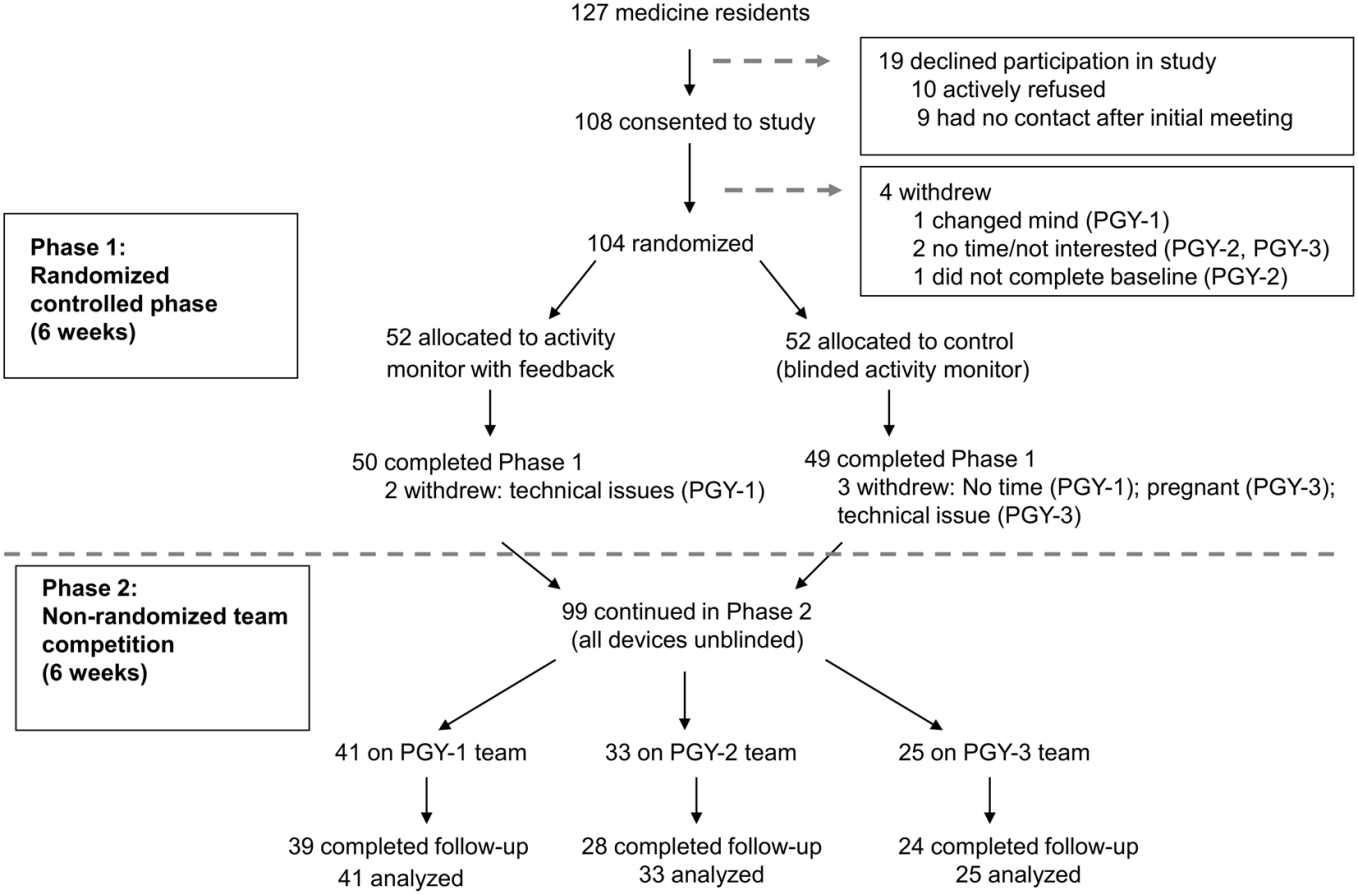
Study flow diagram.

A total of 108 of 127 (85%) internal medicine residents enrolled in the study between July and November 2010 (Figure 1). Four residents withdrew from the study prior to randomization, and 104 (82%) were randomized in January 2011. Five residents withdrew during Phase 1, and 99 continued participation in Phase 2. At baseline, the mean age of residents was 29, 54% were female, 66% were white, and 52% were living with a significant other. The mean body mass index was 24.1 kg/m^2^, and most residents were non-smokers. Half of residents reported that they had gained weight in the previous year. The intervention and control group did not differ significantly in any of these characteristics (Table 1).

**Table 1:** Baseline characteristics of residents.

	Total	Intervention	Control	P value
	(N = 104)	(N = 52)	(N = 52)	
Age, mean (range)	29 (23–37)	29 (23–36)	9 (25–37)	0.45
Sex, female, %	54	51	56	0.69
Race, white, %	66	66	65	0.95
Married/living with significant other, %	52	46	58	0.24
Body mass index, kg/m^2^, mean (range)	24.1 (17.8–35.6)	23.7 (19.4–34.9)	24.6 (17.8–35.6)	0.23
Non-smoker, %	95	92	98	0.16
Reported gaining weight in past year, %	50	52	46	0.49

Table 2 shows the intention to treat analyses with median steps per day and compliance with wearing the activity monitor during the randomized phase (Phase 1). During this phase, there was no difference in the median steps recorded or compliance with wearing the monitor between the intervention (unblinded) and control (blinded) groups. Table 2 also shows a secondary analysis including only days that the monitor was worn, and there was no significant difference in mean steps per day between the intervention and control groups.

**Table 2:** Median steps per day and compliance with wearing activity monitor during the 6-week randomized phase.

	Intervention*	Control†	Difference	P value
	(N = 50)	(N = 49)		
Steps/day, median (IQR)	6369 (1999–8796)	6063 (1299–8723)	306	0.16
Number of days activity monitor worn, mean (%)	33.2 (77%)	33.0 (77%)	0.2	0.73
Steps/day on days monitor worn, mean (SD)	7886 (3622)	7600 (3492)	286	0.63

IQR = interquartile range; SD = standard deviation.

*Residents in intervention group wore an unblinded monitor (with visual activity feedback) during both Phase 1 (randomized) and Phase 2 (team competition).

† Residents in the control group wore a blinded monitor (no feedback) during Phase 1 and an unblinded monitor during Phase 2.

**Figure 2:**
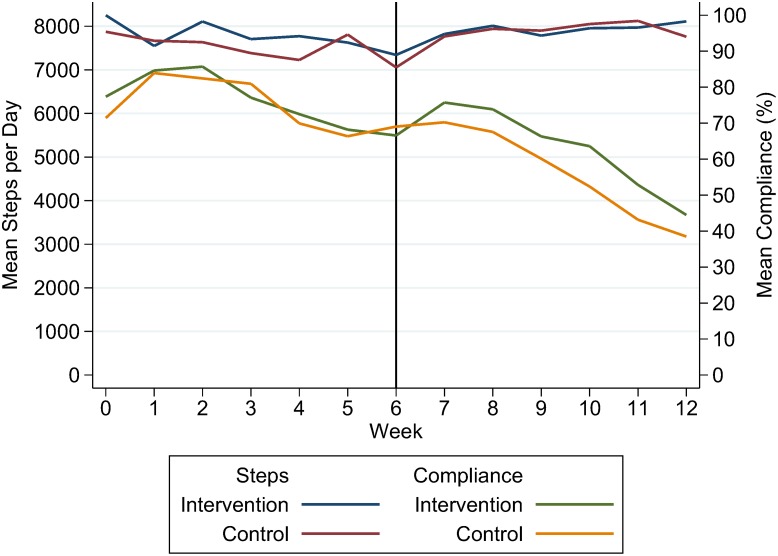
Resident activity levels and compliance with wearing activity monitor by study arm over 12 weeks. Activity levels are the number of steps taken on days when steps were recorded (>500 steps/day). Compliance is the mean percentage of days residents wore the activity monitor per week.

Figure 2 shows the mean steps per day for days when the activity monitor was worn (>500 steps recorded in a day). More steps were recorded during the team competition phase compared to the randomized phase; this was statistically significant for residents assigned to the control group for Phase 1 (7,971 vs. 7,567, p = 0.002) but not for residents assigned to the intervention group (7,832 vs. 7,739, p = 0.13). Mean compliance with wearing the activity monitor decreased for both groups during Phase 2 compared to Phase 1 (60% vs. 77%, p<0.001).

**Figure 3:**
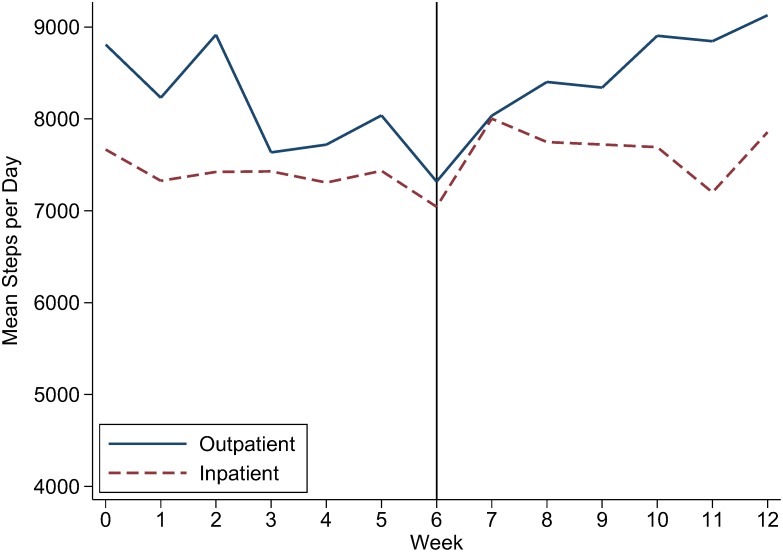
Activity levels during inpatient and outpatient rotations over 12 weeks. Activity levels are the number of steps taken on days when steps were recorded (>500 steps/day) when residents were on inpatient or outpatient rotations.

Figure 3 shows mean steps per day on inpatient or outpatient rotations. Residents recorded a higher mean number of steps per day during outpatient rotations compared to inpatient rotations during the entire study (difference of 648 steps/day, p<0.001).

Table 3 shows the change in body composition, blood pressure, and lipids from baseline to the end of the 12-week study for all residents. At study end, mean systolic blood pressure decreased (p = 0.004) and HDL cholesterol increased (p<0.001), but there were no significant changes in weight, BMI, diastolic blood pressure, or total and LDL cholesterol.

**Table 3:** Change in body composition, blood pressure, and lipids at baseline and end of the study.

	Baseline	End of study	P value
Weight, lbs, mean (SD)*	154 (27.9)	155 (29.8)	0.59
Body mass index, mean (SD)*	23.8 (3.9)	23.9 (4.2)	0.67
Waist, inches, mean (SD)†	30.2 (3.9)	30.2 (3.8)	0.83
Systolic BP, mm Hg, mean (SD)‡	121 (15.4)	117 (12.6)	0.004
Diastolic BP, mm Hg, mean (SD)‡	76 (11.2)	77 (8.3)	0.13
Total cholesterol, mg/dL, mean (SD)§	169 (20.7)	172 (26.5)	0.19
LDL cholesterol, mg/dL, mean (SD)§	94 (22.0)	92 (26.7)	0.44
HDL cholesterol, mg/dL, mean (SD)§	57 (14.7)	61 (15.7)	<0.001

SD = standard deviation; IQR = interquartile range.

*Weight and BMI data available at baseline and end of study for 91 residents.

† Waist and self-reported exercise available at baseline and end of study for 87 residents.

‡ Blood pressure available at baseline and end of study for 81 residents.

§ Fasting cholesterol available at baseline and end of study for 67 residents.

We assessed residents’ utilization of the on-site fitness center, personal training, and individual nutrition appointments offered during the 12 week study. From July–December 2010 (prior to intervention), 3 residents (3%) had used the fitness center. During the study when residents were given a free membership and personal training, 54 residents (55%) used the fitness center at least once (mean visits = 6.7, range 1 to 21), and of these, 42 (76%) went to at least one personal training session (mean sessions = 4.4, range 1 to 10). Twenty-three residents (23%) met with the nutritionist individually.

## Discussion

This is the first randomized study to test an activity monitor to promote physical activity among physicians. Despite their busy schedules, we found that medical residents were motivated to participate in a physical activity program, as evidenced by high consent, participation, and follow up rates. However, wearing an activity monitor with real time feedback did not result in increased activity compared to wearing a blinded monitor. Adding the team competition had a modest effect on increasing activity levels of residents. For all residents, there were significant improvements in systolic blood pressure and HDL cholesterol at the end of the study. Although this activity monitor intervention did not have a major impact on activity or health, the high participation rates of busy residents and modest changes in steps, blood pressure, and HDL suggest that more intensive hospital worksite wellness programs have potential for promoting healthier lifestyles among health care professionals.

The rate of burnout among physicians, especially residents and primary care providers, is higher than for other professions. [41] Although duty hour restrictions will likely contribute to some improvement, [28], [28,42] strengthening physician resilience to burnout, depression, and illness also requires attention to the positive aspects of wellness. Physicians report physical exercise as one of the coping strategies for avoiding burnout and maintaining wellness, [43]–[45] but many physicians do not adequately incorporate regular exercise into their lives. [17], [19]–[21] Our study demonstrates that a wellness program using activity monitors to promote physical activity had a high rate of participation and was feasible to incorporate into a busy internal medicine residency schedule. Future research will be needed to determine whether increasing physical activity and other healthy lifestyle habits of will lead to lower rates of burnout and better health.

This study has limitations. Two factors may have contributed to increased activity levels of both groups, resulting in the finding of no difference in steps during Phase 1. First, we were not able to measure baseline steps per day, and it is possible that the residents in both the intervention and control groups increased their steps because they knew steps were being monitored. Second, free access to the fitness center was provided to residents assigned to both groups. This study included residents from a single residency program in an urban setting, and therefore the results may not be generalizable to all residents and physicians.

Physicians spend a majority of their time at work, and therefore hospital or office-based worksite wellness programs could provide an opportunity to initiate and reinforce healthy habits. Our results clearly show that this young, healthy population of medical residents had activity levels below the recommended 10,000 steps per day, [46] and therefore more intense interventions will be needed for clinically significant health benefits. Although using an activity monitor with real time and on-line feedback did not result in a statistically significant increase in activity, adding a team steps competition did result in a small increase. Residents utilized the on-site gym more frequently during the 12-week program, and there was some evidence of improvement in systolic blood pressure and HDL cholesterol at the end of the study. Despite the perception that residents on inpatient rotations are on their feet walking for extended hours, a secondary analysis revealed that residents recorded more steps when they were on outpatient rotations and had more time for leisure-time physical activity. Increasing opportunities for residents to be physically active during outpatient rotations will promote exercise during training as well as provide a model for maintaining healthy habits post-training.

Promoting healthy lifestyle habits of physicians will not only create better role models for patients but will also improve physician health. Hospitals, residency programs, and other healthcare institutions should consider ways to integrate wellness programs into medical training and practice. The Institute of Medicine suggests that “health care organizations and providers serve as models for the incorporation of healthy eating and active living into worksite practices and programs”. [10] Potential opportunities for increasing physical activity among physicians, especially in the inpatient setting, might include encouraging structured physical activity breaks, allotting space for on-site exercise equipment, and creating team competitions among physicians and other health care professionals. These types of interventions are relatively low cost and may ultimately increase work efficiency and contribute to a healthier and happier physician workforce.

## Supporting Information

Protocol S1 **Trial Protocol.**
(DOCX)

Checklist S1 **CONSORT checklist.**
(DOCX)
