# Supplementary material for: Activity Monitor Intervention to Promote Physical Activity of Physicians-In-Training: Randomized Controlled Trial
Source: PLoS One. 2014 Jun 20;9(6):e100251. doi: 10.1371/journal.pone.0100251 (PMC4065028; doi:10.1371/journal.pone.0100251)
Supplement: Protocol S1 — Trial Protocol. (DOCX) [file pone.0100251.s001.docx]

Answer all questions accurately and completely in order to provide the PHRC with the relevant information to assess the risk-benefit ratio for the study. Do not leave sections blank.

# PRINCIPAL/OVERALL INVESTIGATOR

Anne Thorndike, MD, MPH

# PROTOCOL TITLE

Be Fit in Residency

# FUNDING

Departmental

# VERSION DATE

June 18, 2010

### SPECIFIC AIMS

Concisely state the objectives of the study and the hypothesis being tested.

The main objective of this study is to test an intervention to increase the physical activity of medical residents, an employee population with little time for exercise. Specifically, the aims of this study are:

1. To determine if providing medical residents with an activity device that measures steps, distance, and calories burned and tracks this information over time on a website increases residents’ physical activity levels as measured by number of steps per day compared to a control group using a blinded activity device (no feedback).
2. To determine if an unblinded team competition using the activity device directly following the randomized phase increases residents’ activity level compared to baseline.
3. To determine if activity level is associated with change in weight during the residency year.
4. To determine if the average hours of sleep per week is associated with changes in weight and with activity level.

# BACKGROUND AND SIGNIFICANCE

Provide a brief paragraph summarizing prior experience important for understanding the proposed study and procedures.

Poor diet and physical inactivity accounted for 400,000 deaths in 2000 and will soon overtake tobacco as the leading cause of mortality in the United States. Poor diet and physical inactivity are associated with many chronic diseases, including cardiovascular diseases, diabetes, and cancer, and are associated with high health care costs. The worksite is an ideal setting for promoting physical activity and nutrition because it has already established channels of communication, support networks, and opportunities for developing corporate norms. Health promotion programs in the workplace have been shown to improve cardiovascular risk factors and reduce health care costs.

Massachusetts General Hospital has sponsored an employee physical activity and nutrition program since June 2005. Over 3000 employees have participated in this program since it was first started. However, none of the participants have been medical residents. The residents have been unable to participate in the 12-week program because of their busy clinical schedules. Medical residents have little time for self-care, including nutrition and physical activity. Poor health habits established during residency may contribute to weight gain and sedentary lifestyle long after the residency has been completed. Although there are many studies of resident burnout, there is a lack of studies addressing health promotion among residents.

Pedometers are relatively inexpensive devices usually worn on the hip that measure number of steps walked per day. A systematic review of 26 studies of pedometers concluded that pedometer use is associated with significant increases in physical activity and significant decreases in body mass index and blood pressure. A pedometer can easily be worn in a work environment and could potentially serve as a motivation to be more active while at work.

An activity monitor is similar to a pedometer, but it provides information about not only steps but also about speed and intensity of activities. The objective of the proposed research is to test an activity monitor as a tool to increase medical residents’ physical activity by increasing steps taken both at work and at home.

###### RESEARCH DESIGN AND METHODS

Briefly describe study design and anticipated enrollment, i.e., number of subjects to be enrolled by researchers study-wide and by Partners researchers. Provide a brief summary of the eligibility criteria (for example, age range, gender, medical condition). Include any local site restrictions, for example, “Enrollment at Partners will be limited to adults although the sponsor’s protocol is open to both children and adults.”

All 170 residents in Medicine at Massachusetts General Hospital will be invited to join the Be Fit program during the time period July-November 2010. Be Fit will be a program for the residents that provides nutrition information and counseling, free access to the on-site gym at MGH, and the opportunity to work with a personal trainer. If a resident signs up for Be Fit and completes the baseline physical assessment, he or she will be eligible for participating in the research study of the activity monitor. If a resident does not enroll in Be Fit, he or she is not eligible to participate in the research study. It is anticipated that approximately 100 residents will enroll in the research study.

Briefly describe study procedures. Include any local site restrictions, for example, “Subjects enrolled at Partners will not participate in the pharmacokinetic portion of the study.” Describe study endpoints.

All medicine residents rotate through a 4 week ambulatory block between July and November 2010. During this ambulatory block, each group of approximately 34 residents will be introduced to the Be Fit program and to the research study during a 1 hour session as part of their ambulatory block lecture series. The PI or one of the research staff members will review the consent form during this time with all the residents. Residents will be given the consent form to take home with them if they would like more time to read it over and will be given the opportunity to ask questions about the study. In order to participate in the research study, a resident must sign the consent form prior to the end of November.

Be Fit program:

All medicine residents will be given the opportunity to enroll in the Be Fit program during their ambulatory block rotation between July and November 2010. If a resident signs up for Be Fit, he or she will participate in a baseline assessment. During this assessment, the resident’s height, weight, waist circumference, body fat, blood pressure, and pulse will be measured. The resident will also have fasting blood test for a lipid profile. This same assessment will be repeated in May 2011. All measurements will be summarized in a letter that is emailed to the resident. The letter sent in May will compare the baseline measurements to the end of program measurements.

All residents who enroll in the Be Fit program (but not necessarily the research study) will be given a card for 36 free visits at on-site gym at MGH (Clubs at Charles River Park) between January and June 2011. In addition, the residents will be able to schedule up to 12 hours of personal training at the gym.

The nutrition education will take place during noon conference lunches, which are provided for all MGH medicine residents daily on Monday-Friday. These lunches are ordered by the Department of Medicine from local restaurants. Starting in January 2011, one of the noon lunches each week will be a “Be Fit” lunch that is lower in fat and calories than one of the typical noon lunches (i.e. Indian food, pizza, Thai food). In addition, the Be Fit nutritionists will provide nutrition information for all lunches during the week. For example, the number of calories and fat in one slice of pepperoni pizza or in one serving of pad thai will be posted at the table where the residents get their food. As part of the Be Fit program, residents will be given the opportunity to meet individually with a nutritionist twice during the period between January and June 2011.

Research study:

Residents who sign the informed consent will participate in the research study starting in January 2011. The research study will consist of 2 phases. In the first phase, half of the study subjects will be randomized to wearing an activity monitor with feedback, and half will be randomized to wearing an activity monitor with no feedback (blinded/control group). This phase will last for 6 weeks. Immediately following the randomized phase, all subjects will be unblinded and wear the activity monitor with feedback. The second phase will be a team competition between the 3 classes of residents (interns, juniors, and seniors). The teams will compete on average number of steps per week for each team. The team competition will last for 6 weeks.

Randomization: Study participants will be randomized in late December to either the intervention arm (activity monitor with feedback) or the control arm (activity monitor with no feedback). The randomization will be stratified by class year (intern, junior, senior).

Parameters to be measured:

The parameters that will be measured for all Be Fit program participants at baseline and at the end of the program are listed in the table below. The survey will collect data on health habits, including smoking, nutrition, physical activity, and sleep.

|  | Baseline  (July-November 2010) | End of program  (May 2011) |
| --- | --- | --- |
| **Physical assessment:** Blood pressure and heart rate, weight, BMI, body fat, waist circumference | X | X |
| **Blood draw:** Fasting lipids | X | X |
| Survey (completed by participant) | X | X |

For those Be Fit participants who enroll in the research study, we will collect data measured by the activity device worn from January through May 2011. The activity device records data on number of steps per day, number of calories burned per day, number of hours slept per night, and number of times awakened per night.

Device to be used: The “Fitbit”: Each study subject will be given a Fitbit, an activity monitor that wirelessly uploads data through a receiving device connected to the subject’s home computer. The activity monitor will be purchased from Fitbit, Inc, and ambulatory monitoring technology company. The Fitbit Tracker contains a motion sensor like the ones found in the Nintendo Wii, and it senses motion in three dimensions. The Tracker measures the intensity and duration of physical activities, calories burned, steps taken, distance traveled, how long it takes to fall asleep, and the amount of time asleep. The Fitbit is worn on the torso, including the waist or chest. It can also be clipped to an undergarment or carried in a pocket. The Fitbit needs to be charged every 5-10 days on a base station that plugs into a computer. It typically takes 60-90 minutes to fully charge. Each study subject will be assigned an anonymous gmail account and a study identification number that will be used to set up the personal Fitbit account. The key linking the subject’s name to the gmail account and the study ID will be stored in a file on the principal investigator’s Partners-protected computer. Study subjects will be able to access their personal Fitbit website by entering the gmail address and the study ID.

Randomized phase: Half of the subjects will be randomized to wearing the Fitbit with feedback. These subjects will be able to see the number of steps walked, calories burned, and distance traveled for each day. On the Fitbit website, the subject will have access to a personalized Fitbit website to view his or her daily and weekly totals of all measurements. Subjects randomized to the control group will wear the activity device but will not be able to see any of the measurements on the device or on the website. Subjects in the control group will receive email reminders to charge their Fitbit since they will not be able to view the battery icon on the device while it is blinded. The randomized phase will last for 6 weeks.

Team competition phase: Immediately following the randomization phase, all study participants will be unblinded and able to view the Fitbit feedback both on the device and on the website. The control group will be given a password to be able to log on to their personalized Fitbit website. The residents will be divided into 3 teams based on their class: interns, juniors, or seniors. The teams will compete on the average number of steps per week. The team standings will be displayed on the Fitbit website that will be viewed by each study subject. Teams will compete for 6 weeks.

Data to be collected:

Physical assessment*:* All residents who enroll in the Be Fit program will be asked to attend a physical assessment at baseline (between July and November 2010) and at the end (May 2011). All physical assessments will be conducted prior to 10 o’clock in the morning. During these visits, study staff collect weight, height, waist measurements, body fat, blood pressure, and heart rate.

Fasting blood test*:* During each physical assessment, residents enrolled in Be Fit undergo a fasting blood test for lipids. Residents are asked not to have anything to eat or drink after midnight the day before the test.

Survey: All residents enrolled in Be Fit will complete a survey at baseline and at the end of the program. The survey collects information on smoking history, physical activity, and nutritional habits. Age, sex, race/ethnicity, marital status, education level, and care for dependents at home (children, disabled/ill spouse, parent, or child) will be recorded on the survey. The survey also includes a scale that measures psychological stress, called the Schwartz Outcome Scale-10 © (SOS-10 TM). The SOS-10 is a 10 item survey tool to determine a person’s distress level. It is not a diagnostic instrument and cannot be used for making psychiatric diagnoses. The scores range from 0 to 60. Higher scores reflect better psychological health and lower scores indicate emotional distress and lower levels of psychological health. There is a cut off score of 43, and a change of 8 points or greater are a reliable indicator that a person’s psychological status has either improved or deteriorated.

Data from Fitbit website: Only residents who enroll in the research study will be given a Fitbit activity device. All activity data and sleep data recorded during the study period will be collected. The Fitbit website provides individual data on the number of steps per day, the number of calories burned per day, and the number of hours slept per 24 hour period. This data will be collected from all study subjects during both the randomized phase and the team competition phase of the study.

Study endpoints:

Primary endpoint: The primary endpoint of the study will be average activity level per resident as measured by number of steps per day. In the randomized phase, the average number of steps per day in the intervention group (Fitbit + feedback) will be compared to the average number of steps per day in the control group (Fitbit without feedback). In the second phase, we will compare the change in the average number of steps for each participant compared to their own baseline number of steps from the first phase.

Secondary endpoints: Secondary endpoints will be change in weight during the study period and number of hours of sleep per 24 hour period for each resident. Survey data will be used to examine reported changes in physical activity levels, nutritional habits, and sleep habits. The survey data will also provide data from the SOS-10 scale on change in psychological stress.

Statistical methods:

In the randomized phase, the average number of steps per day in the intervention group (Fitbit + feedback) will be compared to the average number of steps per day in the control group (Fitbit without feedback) using a two-sample t test or a Wilcoxon rank sum test whichever is more appropriate. To determine whether team competition increases activity level, the primary and secondary endpoints from the second phase will be compared to results from the first phase using paired t-tests. The changes in primary and secondary endpoints during study periods will be compared between the two randomization groups using a two-sample t test, and compared among the residency year groups using an analysis of variance approach. To determine if the average hours of sleep per week is associated with changes during study period, Pearson or Spearman correlation coefficients will be used to summarize the correlation between sleep time and change in weight/number of steps.

Power analysis: For the randomization phase, a sample size of 50 in each group will have 84% power to detect a difference in means of 1200 steps assuming that the common standard deviation is 2000 steps using a two group t-test with a 0.05 two-sided significance level.

For studies involving treatment or diagnosis, provide information about standard of care at Partners (e.g., BWH, MGH) and indicate how the study procedures differ from standard care. Provide information on available alternative treatments, procedures, or methods of diagnosis.

This study does not involve treatment or diagnosis of a medical condition or illness.

Describe how risks to subjects are minimized, for example, by using procedures which are consistent with sound research design and which do not unnecessarily expose subjects to risk or by using procedures already being performed on the subject for diagnostic or treatment purposes.

The risk associated with this research is minimal. The principle investigator oversees all study staff to ensure that procedures are followed which are consistent with sound research design and do not expose subjects to unnecessary risk. The study does not offer a diagnosis or treatment to subjects. The procedures in study were developed using standard nutrition and fitness guidelines and recommendations.

Describe explicitly the methods for ensuring the safety of subjects. Provide objective criteria for removing a subject from the study, for example, objective criteria for worsening disease/lack of improvement and/or unacceptable adverse events. The inclusion of objective drop criteria is especially important in studies designed with placebo control groups.

Subjects will be withdrawn from the study if they leave their employment at MGH or if they request to be removed from the study.

# FORESEEABLE RISKS AND DISCOMFORTS

Provide a brief description of any foreseeable risks and discomforts to subjects. Include those related to drugs/devices/procedures being studied and/or administered/performed solely for research purposes. In addition, include psychosocial risks, and risks related to privacy and confidentiality. When applicable, describe risks to a developing fetus or nursing infant.

The risk for this study is minimal. The study consists of wearing an activity monitor that wirelessly uploads activity and sleep data to a website . Every resident who participates in the Be Fit program has 2 physical assessments and blood tests and fills out 2 surveys. If a resident is participating in the research study, their data will be included in the research database. Psychosocial and privacy risks are also estimated to be minimal. Confidentiality will be ensured by de-identifying subjects’ data with study identification numbers.

All study subjects will receive an activity monitor. This device does not pose any medical risk and does not diagnosis any medical conditions or illnesses. Wearing the activity monitor may cause minimal discomfort. The device is small, weighing only a few ounces, and can be easily worn on the torso by attaching it to a belt or waistband, pants, a shirt, or an undergarment.

**EXPECTED BENEFITS**

# Describe both the expected benefits to individual subjects participating in the research and the importance of the knowledge that may reasonably be expected to result from the study. Provide a brief, realistic summary of potential benefits to subjects, for example, “It is hoped that the treatment will result in a partial reduction in tumor size in at least 25% of the enrolled subjects.” Indicate how the results of the study will benefit future patients with the disease/condition being studied and/or society, e.g., through increased knowledge of human physiology or behavior, improved safety, or technological advances.

Study participants may not receive any direct benefit from being in the research study. It is hoped that participation in the intervention will help subjects increase their activity levels and therefore improve or prevent clinical risk factors that are associated with disease.

The results of this research may provide better understanding of how to help medical residents and other employees increase their activity levels, and if these changes are associated with improved sleeping habits and prevention of weight gain. The results could also inform other institutions about the benefits of establishing similar programs.

**EQUITABLE SELECTION OF SUBJECTS**

The risks and benefits of the research must be fairly distributed among the populations that stand to benefit from it. No group of persons, for example, men, women, pregnant women, children, and minorities, should be categorically excluded from the research without a good scientific or ethical reason to do so. Please provide the basis for concluding that the study population is representative of the population that stands to potentially benefit from this research.

All MGH Medicine residents are eligible to participate in the Be Fit program. Children will be excluded because medicine residents are typically at least 24 years old. Otherwise, no group of men, women, pregnant women, or minorities will be excluded from the study.

When people who do not speak English are excluded from participation in the research, provide the scientific rationale for doing so. Individuals who do not speak English should not be denied participation in research simply because it is inconvenient to translate the consent form in different languages and to have an interpreter present.

All MGH medicine residents are proficient in English.

For guidance, refer to the following Partners policy:

Obtaining and Documenting Informed Consent of Subjects who do not Speak English

[**http://healthcare.partners.org/phsirb/nonengco.htm**](http://healthcare.partners.org/phsirb/nonengco.htm)

**RECRUITMENT PROCEDURES**

Explain in detail the specific methodology that will be used to recruit subjects. Specifically address how, when, where and by whom subjects will be identified and approached about participation. Include any specific recruitment methods used to enhance recruitment of women and minorities.

Every Medicine resident rotates through a 4 week ambulatory block between July and November 2010. During this ambulatory block, each group of approximately 34 residents will be introduced to the Be Fit program and to the research study during a 1 hour session as part of their ambulatory block lecture series. If a resident chooses to participate in the Be Fit program, he or she will be asked to participate in the research study.

Provide details of remuneration, when applicable. Even when subjects may derive medical benefit from participation, it is often the case that extra hospital visits, meals at the hospital, parking fees or other inconveniences will result in additional out-of-pocket expenses related to study participation. Investigators may wish to consider providing reimbursement for such expenses when funding is available

To increase compliance with wearing the device on a regular basis, we will offer a small gift certificate at the hospital coffee shop as an incentive. All residents who wear the device regularly will be eligible each week to be entered into a raffle for the gift certificate. At the end of each week, we will calculate the number of days each resident has worn their Fitbit. All residents who have worn their Fitbit for 5 of the previous 7 days will be put into a raffle to win a $10 gift certificate for Coffee Central at MGH. The winner each week will be announced in the weekly email to all the study participants. We will conduct this raffle once a week for the entire 12 week study period.

At the end of the study, If the participant wears the Fitbit activity device for 75% of the intervention period (12 weeks), the study participant will be allowed to keep the device (retail value of $99.00) for their own personal use.

For guidance, refer to the following Partners policies:

Recruitment of Research Subjects

[**http://healthcare.partners.org/phsirb/recruit.htm**](http://healthcare.partners.org/phsirb/recruit.htm)

Guidelines for Advertisements for Recruiting Subjects

[**http://healthcare.partners.org/phsirb/advert.htm**](http://healthcare.partners.org/phsirb/advert.htm)

Remuneration for Research Subjects

[**http://healthcare.partners.org/phsirb/remun.htm**](http://healthcare.partners.org/phsirb/remun.htm)

#### CONSENT PROCEDURES

Explain in detail how, when, where, and by whom consent is obtained, and the timing of consent (i.e., how long subjects will be given to consider participation). For most studies involving more than minimal risk and all studies involving investigational drugs/devices, a licensed physician investigator must obtain informed consent. When subjects are to be enrolled from among the investigators’ own patients, describe how the potential for coercion will be avoided.

The PI will review the consent form during the 1 hour ambulatory block lecture with all the residents in the room. There will be 5 different 1 hour sessions with approximately 34 residents in attendance at each session. If the PI is not available for one of these sessions, a member of the research staff will review the consent form. Residents will be given the consent form to take home with them if they would like more time to read it over and will be given the opportunity to ask questions about the study. If a resident is not able to attend the 1 hour session, the PI or another study staff member will review the consent form with the resident individually. In order to participate in the research study, a resident must sign the consent form prior to the end of November 2010.

NOTE: When subjects are unable to give consent due to age (minors) or impaired decision-making capacity, complete the forms for Research Involving Children as Subjects of Research and/or Research Involving Individuals with Impaired Decision-making Capacity, available on the New Submissions page on the PHRC website:

[**http://healthcare.partners.org/phsirb/newapp.htm#Newapp**](http://healthcare.partners.org/phsirb/newapp.htm#Newapp)

For guidance, refer to the following Partners policy:

Informed Consent of Research Subjects

[**http://healthcare.partners.org/phsirb/infcons.htm**](http://healthcare.partners.org/phsirb/infcons.htm)

## DATA AND SAFETY MONITORING

Describe the plan for monitoring the data to ensure the safety of subjects. The plan should include a brief description of (1) the safety and/or efficacy data that will be reviewed; (2) the planned frequency of review; and (3) who will be responsible for this review and for determining whether the research should be altered or stopped. Include a brief description of any stopping rules for the study, when appropriate. Depending upon the risk, size and complexity of the study, the investigator, an expert group, an independent Data and Safety Monitoring Board (DSMB) or others might be assigned primary responsibility for this monitoring activity.

NOTE: Regardless of data and safety monitoring plans by the sponsor or others, the principal investigator is ultimately responsible for protecting the rights, safety, and welfare of subjects under his/her care.

This research study utilizes a minimal risk intervention. We do not expect adverse effects or safety concerns, nor do we anticipate the need for the Data Safety Monitoring Board to review this application.

Describe the plan to be followed by the Principal Investigator/study staff for review of adverse events experienced by subjects under his/her care, and when applicable, for review of sponsor safety reports and DSMB reports. Describe the plan for reporting adverse events to the sponsor and the Partners’ IRB and, when applicable, for submitting sponsor safety reports and DSMB reports to the Partners’ IRBs. When the investigator is also the sponsor of the IND/IDE, include the plan for reporting of adverse events to the FDA and, when applicable, to investigators at other sites.

NOTE: In addition to the adverse event reporting requirements of the sponsor, the principal investigator must follow the Partners Human Research Committee guidelines for Adverse Event Reporting

The PI will review all blood test results obtained from Be Fit participants to screen for abnormalities. She will review any adverse events or subject complaints, including any breech of confidentiality or privacy, and these will be reported to the Partners IRB in the annual continuing review. Any serious adverse events will be reported to the Partners’ IRB within 24 hours.

## MONITORING AND QUALITY ASSURANCE

Describe the plan to be followed by the principal investigator/study staff to monitor and assure the validity and integrity of the data and adherence to the IRB-approved protocol. Specify who will be responsible for monitoring, and the planned frequency of monitoring. For example, specify who will review the accuracy and completeness of case report form entries, source documents, and informed consent.

NOTE: Regardless of monitoring plans by the sponsor or others, the principal investigator is ultimately responsible for ensuring that the study is conducted at his/her investigative site in accordance with the IRB-approved protocol, and applicable regulations and requirements of the IRB.

The PI will ensure that this study is conducted in accordance with all IRB rules and regulations by maintaining regular contact with study staff. Collectively, they will conduct routine study audits to confirm that all study materials and data are following study procedures as outlined in the IRB approved study protocol.

For guidance, refer to the following Partners policies:

##### Data and Safety Monitoring Plans and Quality Assurance

[**http://healthcare.partners.org/phsirb/datasafe.htm**](http://healthcare.partners.org/phsirb/datasafe.htm)

Adverse Event Reporting Guidelines

**<http://healthcare.partners.org/phsirb/adverse_events.htm>**

# PRIVACY AND CONFIDENTIALITY

Describe methods used to protect the privacy of subjects and maintain confidentiality of data collected. This typically includes such practices as substituting codes for names and/or medical record numbers; removing face sheets or other identifiers from completed surveys/questionnaires; proper disposal of printed computer data; limited access to study data; use of password-protected computer databases; training for research staff on the importance of confidentiality of data, and storing research records in a secure location.

NOTE: Additional measures, such as obtaining a Certificate of Confidentiality, should be considered and are strongly encouraged when the research involves the collection of sensitive data, such as sexual, criminal or illegal behaviors.

Confidentiality and protecting employees’ information are priorities. Study staff will follow standard practices of research to protect the privacy of all participants and to maintain confidentiality. The study database manager (Sue Regan, PhD) has created a separate research database on the Partners secure server in a Shared File Area (SFA). Only study staff will have access to this SFA as granted by the study staff “key giver” (Sue Regan). Names of the study participants will not appear in this research study database. Participants’ identifiers will be removed and replaced with unique study identification numbers. The key that links participants’ names with identification numbers will exist in the Be Fit program database on a separate SFA.

All study surveys will be given to subjects to complete in paper format. The data will be entered into the study database by a member of the study staff. The paper copies of the survey will be maintained in a locked file cabinet in the office of the PI for one year. After this time, the survey will be properly destroyed with a shredder. Study participants’ data collected from the Fitbit website will be downloaded weekly by the database manager. This data will be entered into the study database under the participants’ study identification number. A Business Associates Agreement will be signed by Fitbit to ensure that they will uphold the privacy standards set by HIPAA.

SENDING SPECIMENS/DATA TO RESEARCH COLLABORATORS OUTSIDE PARTNERS

Specimens or data collected by Partners investigators will be sent to research collaborators outside Partners, indicate to whom specimens/data will be sent, what information will be sent, and whether the specimens/data will contain identifiers that could be used by the outside collaborators to link the specimens/data to individual subjects.

Specimens and data will not be sent to research collaborators outside of Partners.

Specifically address whether specimens/data will be stored at collaborating sites outside Partners for future use not described in the protocol. Include whether subjects can withdraw their specimens/data, and how they would do so. When appropriate, submit documentation of IRB approval from the recipient institution.

Specimens and data will not be stored at collaborating sites outside of Partners.

# RECEIVING SPECIMENS/DATA FROM RESEARCH COLLABORATORS OUTSIDE PARTNERS

When specimens or data collected by research collaborators outside Partners will be sent to Partners investigators, indicate from where the specimens/data will be obtained and whether the specimens/data will contain identifiers that could be used by Partners investigators to link the specimens/data to individual subjects. When appropriate, submit documentation of IRB approval and a copy of the IRB-approved consent form from the institution where the specimens/data were collected.

This study will not be receiving specimens from outside of Partners.
